# The Office Hour

**Published:** 1897-08

**Authors:** 


### The Office Hour.

A shrewd, capable, and eminently successful physician once said that his success was largely due to a motto, "Take care of your office hour and your office hour will take care of you." When the large amount of waste time in the average physician's routine, the hour's drive and the ten-minute call, is considered, one can readily appreciate the business importance of so regulating work as to reduce this waste to a minimum. Men have long ago learned that in commercial matters the only people worth dealing with are those whose business goes to them. The peddler or agent who comes to our house is, on the whole, an inconvenience, and his wares or his investments are proverbially defective or expensive. There are many patients physically unable to leave their homes; these must be visited without regard to preference or waste of time in transit. But when patients, physically able to go to the doctor's office, and, perhaps, even needing exercise, prefer to summon him to their homes, something is wrong.

If one intends to build up an office practice, he must perform his part of the implied contract and be on hand to keep his appointment, even if temporary advantage is promised from breaking it. Manifestly, however, a really urgent case must take precedence of one which will suffer only inconvenience from not finding the physician.

The office should be cheerful and wholesome. There should be a happy mean between the appearance, that of being a smoking-room and "den" for the physician, or a play-room for his children, and the worse extreme of emphasizing the horrors of operation and the unpleasant details of anatomy.—*Medical and Surgical Reporter.*

